# Deformability in Unsaturated Polyester Resin-Based Concrete: Effects of the Concentration of Shrinkage-Reducing Agent and Type of Filler

**DOI:** 10.3390/ma13030727

**Published:** 2020-02-05

**Authors:** Jung Heum Yeon, Hee Jun Lee, Jaeheum Yeon

**Affiliations:** 1 Department of Civil & Environmental Engineering, Gachon University, Seongnam, Gyeonggi 13120, Korea; jyeon@gachon.ac.kr; 2 Department of Regional Infrastructure Engineering, Kangwon National University, Chuncheon, Gangwon 24341, Korea; junnblossom@gmail.com; 3 Department of Engineering and Technology, Texas A&M University-Commerce, Commerce, TX 75429, USA

**Keywords:** unsaturated polyester polymer concrete, unsaturated polyester resin, deformability, shrinkage reduction agent content, filler type

## Abstract

In this study, the effects of shrinkage reduction agent (SRA) content and filler type on the deformability characteristics of unsaturated polyester (UP) resin-based polymer concrete were experimentally investigated. Specifically, the setting shrinkage, thermal expansion, maximum compressive strain and the modulus of elasticity of UP polymer concrete were all analyzed. Setting shrinkage was found to be influenced by the UP resin, the SRA and filler. The thermal expansion, maximum compressive strain and modulus of elasticity were also affected by the aggregate. The effect of SRA content on deformability was found to be greater than that of the filler type. To put UP polymer concrete to efficient use, it is essential to secure proper deformability according to the intended purpose. At that time, it is desirable that the deformation characteristics resulting from the SRA content and filler type sufficiently reflect when the mix proportion is determined. The effects of filler type on the deformability of UP polymer concrete are such that: A uniform dispersion of filler particles impacts the setting shrinkage; the thermal expansion is influenced by the filler’s various thermal expansion properties; the compressive strain is related to the nature of the small spherical particles that tend to fill porosity, producing better packing of the aggregate materials; and the modulus of elasticity is influenced by the density, which is related to the strength of the filler. However, additional in-depth studies are required on all of these elements.

## 1. Introduction

Polymer concrete (PC) is a composite material produced with polymeric binders instead of the cementitious binders found in ordinary Portland cement concrete [1]. Hence, PC consists of well-graded, inorganic aggregates and polymeric binders, instead of the water and cementitious binders typically employed to produce Portland cement concrete [2]. The representative advantages of PC are its high strength, excellent durability (e.g., freeze–thaw, acid and abrasion resistance), very low permeability and fast curing time [3]. The high strength and durability of PC allows for the production of high-quality, precast products with very thin cross-sections and low coverage depths (in reinforced concrete), reducing dead loads in structures and minimizing transportation and build costs [4,5].

PC is employed in a wide range of civil and structural applications, such as bridge decking, pavement overlays, hazardous waste containers, sewer pipes and decorative construction panels [6]. PC is the preferred material for repairing damage to old cementitious concrete structures, because the bonding at the contact surface between the old concrete and polymer material is remarkably good [4]. PC is also employed when building precise structures, due to its superior damping ratio, high adhesion and fast curing [7].

Various polymer binders have been employed with aggregates to produce PC, such as epoxy (EP), unsaturated polyester (UP) and vinyl ester (VE) resins, as well as methyl methacrylate monomer (MMA). Among these binders, the most common polymeric binder for PC is UP resin [1,8]. UP resin is a category of thermosetting polymer that is widely applied in various industrial applications, such as composites, automotive paints, protective coatings, storage tanks, piping and construction [9]. This widespread application of UP resin is due to its convenient processing, structural stability and superb chemical resistance. It was these characteristics that caused UP resin to become popular for PCs [10,11,12].

Despite these advantages, UP resin-based polymer concrete (UP polymer concrete) shows substantial shrinkage during the curing process, as compared to what is seen with ordinary Portland cement concrete [1], resulting in considerable volume change [13]. In addition, the coefficient of thermal expansion is more than twice that of ordinary Portland cement concrete [14]. In other words, poor thermal resistance and heavy dependence on temperature are disadvantageous and undesirable properties of the polymer matrix [1]. Moreover, the modulus of elasticity of UP polymer concrete is remarkably low [1,14], which causes greater deformation than that which occurs with normal Portland cement concrete.

Thus, UP polymer concrete is not desirable in terms of dimensional stability. In order to reduce the setting shrinkage, a shrinkage reduction agent (SRA) can be incorporated as an additive when the PC is produced. In particular, the addition of fillers improves the dimensional stability of the PC and minimizes the temperature dependence of the mechanical properties [5]. Based on these facts, this study experimentally investigated the effects of SRA dosage and filler type on the short-term deformability of UP polymer concrete.

## 2. Significance of the Research

Previous studies on UP polymer concrete have focused on research associated with the material’s physical and mechanical properties [13,15,16,17] or chemical resistance and durability [5,13,14,18,19]. However, little work has addressed its deformability, which is a disadvantage of UP polymer concrete and an important parameter for precast products and onsite applications.

In particular, the novelty of the present study is that the setting shrinkage, thermal expansion and the stress–strain relationship were all investigated. There are currently no studies on the concentration of SRAs and fillers, which are important parameters for the deformation of UP polymer concrete. In the use of fillers, some research has employed fly ash [5,6,7,16], calcium carbonate [4,13], or blast furnace slag micro-fillers [17]. However, few have compared the effectiveness of the use of multiple fillers simultaneously employed to produce concrete.

Thus, this study identifies whether the uses of SRAs and certain fillers effectively improve the dimensional stability of UP polymer concrete.

## 3. Experiment

### 3.1. Materials

#### 3.1.1. Resin and Initiator

Two component systems (i.e., UP resin and an initiator) of UP binders were used to produce the PC. The UP resin employed in this study was of a pre-accelerated, orthophthalic type; the catalyst and its properties are shown in Table 1.

The initiator applied with the UP resin was methyl ethyl ketone peroxide (MEKPO, KeumJung Co., Ltd., Ulsan, Korea), containing 45% dimethyl phthalate (DMP, Aekyung Chemical Co., Ltd., Cheong Yang, Chungcheongnam-do, Korea); its properties are listed in Table 2.

#### 3.1.2. Shrinkage-Reducing Agent

About 6% to 12% volumetric shrinkage occurs when pure UP resin is cured without a filler. However, the amount of shrinkage is reduced when the material is employed to produce PC [20]. Though the amount of its shrinkage is reduced, it still is greater than the amount of shrinkage seen with Portland cement concrete. This greater setting shrinkage is problematic, because it can cause cracking on the surface of the structure, disrupting its dimensional stability. A styrene-based SRA was used to reduce shrinkage in the present study; its properties are shown in Table 3.

#### 3.1.3. Filler

When in a paste form, fillers fill voids between the surfaces of the aggregate. UP binders are liquid. A moisture content of less than 0.5%, no impurities, and low absorption are all required components of a filler [1].

**Figure 1:**
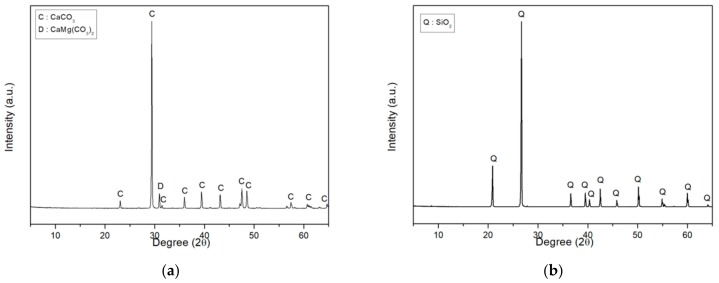
X-ray diffraction (XRD) spectra of the crystalloid fillers: (**a**) Ground calcium carbonate; (**b**) Silica flour.

**Figure 2:**
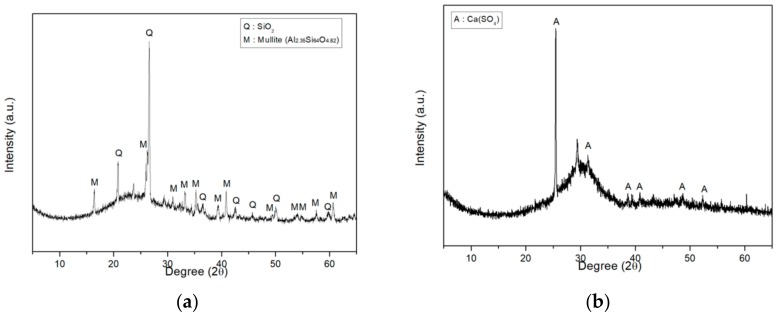
XRD spectra of the amorphous fillers: (**a**) Fly ash; (**b**) Blast furnace slag.

In this study, four types of fillers were employed. These fillers can be categorized into either crystalloid or blast and amorphous fillers. Ground calcium carbonate (GCC) and silica flour (SF) were used as crystalloid fillers. Fly ash (FA) and blast furnace slag (BFS) were employed as amorphous fillers. Density was tested via ASTM D 7481-18: Standard Test Methods for Determining Loose and Tapped Bulk Densities of Powders using a Graduated Cylinder [21]. Particle size and chemical composition were analyzed by LS 13 320 (Beckman Coulter Inc., Brea, CA, USA) particle size analyzer and X-ray fluorescence (XRF, Rigaku Co., Akishima, Tokyo, Japan), respectively. The properties of these fillers are shown in Table 4 and Table 5. Their X-ray diffraction (XRD, Rigaku Co., Akishima, Tokyo, Japan) spectra are shown in Figure 1 and Figure 2.

#### 3.1.4. Aggregate

River sand, gravel, crushed sand and stone can all be used as aggregates. The requirements for aggregates include suitable particle shape, gradation, good soundness, high strength, a moisture content of less than 0.5%, low absorption of binders, etc. [1]. For this study, crushed siliceous aggregates were employed. Their properties were obtained from Gyeongin Materials (Gyeongin Materials Inc., Gimpo, Gyeonggi-do, Korea), and are shown in Table 6.

### 3.2. Preparation of Specimen

#### 3.2.1. Mix Design

Determining the effective mix proportions is essential to reduce the production cost of PC and ensure its structural performance (e.g., high strength and excellent durability). To determine the most effective mix proportions, the basic properties of the PC, such as workability, bleeding or segregation, and strength, should be evaluated. 

The mix proportions of PC depend upon many parameters, such as binder type, shape and gradation of the aggregate, temperature at the worksite, etc. Standards for mix design exist for cement concrete mixtures. However, such standards do not exist for PC. Thus, the mix proportions of PC are generally determined by trial and error. Hence, uniform mix proportions do not exist, and the mix proportions employed in every study are different. The mix proportions applied in the present study are shown in Table 7.

#### 3.2.2. Specimen Preparation

A forced mixing-type mixer was used to produce the specimens of PC because of the fast hardening reaction and high viscosity of the binder. In the mix order, after their weights were measured, the aggregates and fillers were dry-mixed using the mixer. Next, a binder was produced by mixing a resin, SRA, and the initiator, with prepared aggregates and fillers. At that time, the rotational speed of the mixer was set to 60 ± 5 rpm. 

**Figure 3:**
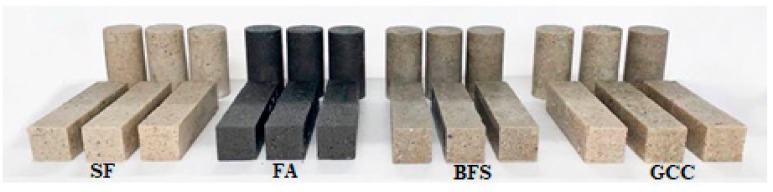
Specimen shape, depending on the type of filler; silica flour (SF), fly ash (FA), blast furnace slag (BFS), and ground calcium carbonate (GCC).

To evenly compact the concrete specimens, hand rodding was carried out for each section. The total height of the cylindrical specimens (Φ 50 × 100 mm) was divided into three sections. The total height of the prismatic specimens (40 × 40 × 160 mm) was evenly divided into two sections. After that, each specimen was put onto a vibrating table (3000 rpm) for three minutes for additional compaction. The specimens were cured at a temperature of 25 ± 2 °C and a humidity of 60 ± 5%. The areas where the load would be applied to the cylindrical specimens were ground for planeness before the compressive stress–strain tests were carried out. The shape of the specimens used in this study is shown in Figure 3. For each test, three test specimens were produced from each batch. 

### 3.3. Test Methods

#### 3.3.1. Setting Shrinkage Test

Setting shrinkage tests were carried out based on ASTM C596-18: Standard Test Method for Drying Shrinkage of Mortar Containing Hydraulic Cement [22]. The size of each test specimen was 40 × 40 × 160 mm. These specimens were cured for seven days in an environmental chamber at a temperature of 23 ± 2 °C and a relative humidity of 60 ± 5%. Shrinkage strain was measured using an embedded-type strain gauge (PMFL-60-5LT, Tokyo Measuring Instruments Laboratory Co., Ltd., Shinagawa, Tokyo, Japan) with a length of 60 mm and a data logger (TDS-602, Tokyo Measuring Instruments Laboratory Co., Ltd, Shinagawa, Tokyo, Japan).

#### 3.3.2. Linear Thermal Expansion Test

Thermal expansion tests were carried out per ASTM C531–18: Standard Test Method for Linear Shrinkage and Coefficient of Thermal Expansion of Chemical Resistance Mortars, Grouts, Monolithic Surfacings, and Polymer Concretes [23]. The size of each test specimen was 40 × 40 × 160 mm. As in the shrinkage test, these specimens were cured for seven days in an environmental chamber at a temperature of 23 ± 2 °C and a relative humidity of 60 ± 5%. A constant-temperature oven was used for the test, and the heating temperature ranged from 25 to 60 °C. The temperature was increased by 5 °C each minute. Also, as with the thermal strain test, thermal expansion was measured using an embedded-type strain gauge (PMFL-60-5LT, Tokyo Measuring Instruments Laboratory Co., Ltd., Shinagawa, Tokyo, Japan) with a length of 60 mm and a data logger (TDS-602, Tokyo Measuring Instruments Laboratory Co., Ltd, Shinagawa, Tokyo, Japan).

#### 3.3.3. Stress-Strain Test

The modulus of elasticity was measured according to ASTM C 469M–14: Standard Test Method for Static Modulus of Elasticity and Poisson’s Ratio of Concrete in Compression [24]. A wire strain gauge with a length of 30 mm was attached to a cylindrical test specimen (Φ 50 × 100 mm). The strain was then measured using a data logger (TDS-602, Tokyo Measuring Instruments Laboratory Co., Ltd, Shinagawa, Tokyo, Japan). A constant rate of loading (0.25 MPa/s) was applied using a universal testing machine of 50 ton. A temperature of 23 ± 2 °C and a relative humidity of 60 ± 5% were maintained for the test space as the compressive test was carried out.

## 4. Results and Discussion

### 4.1. Setting Shrinkage and Thermal Expansion

UP polymer concrete offers many advantages, as mentioned above. However, the material also has certain disadvantages (such as setting shrinkage and thermal expansion), as compared to conventional Portland cement concrete. Nevertheless, studies of UP polymer concrete are rarely carried out to resolve these disadvantages. Further research on the substantial setting shrinkage and thermal expansion of UP polymer concrete should be conducted because these significant strains influence the dimensional stability of a structure. If the setting shrinkage and thermal expansion are repeated as the temperature changes, this could cause interface cohesion failure or shear failure in the substrate, or even loss of broadcast aggregates in the polymer [25,26]. Thus, this study endeavored to investigate the setting shrinkage and the thermal expansion of UP polymer concrete.

#### 4.1.1. Setting Shrinkage

**Figure 4:**
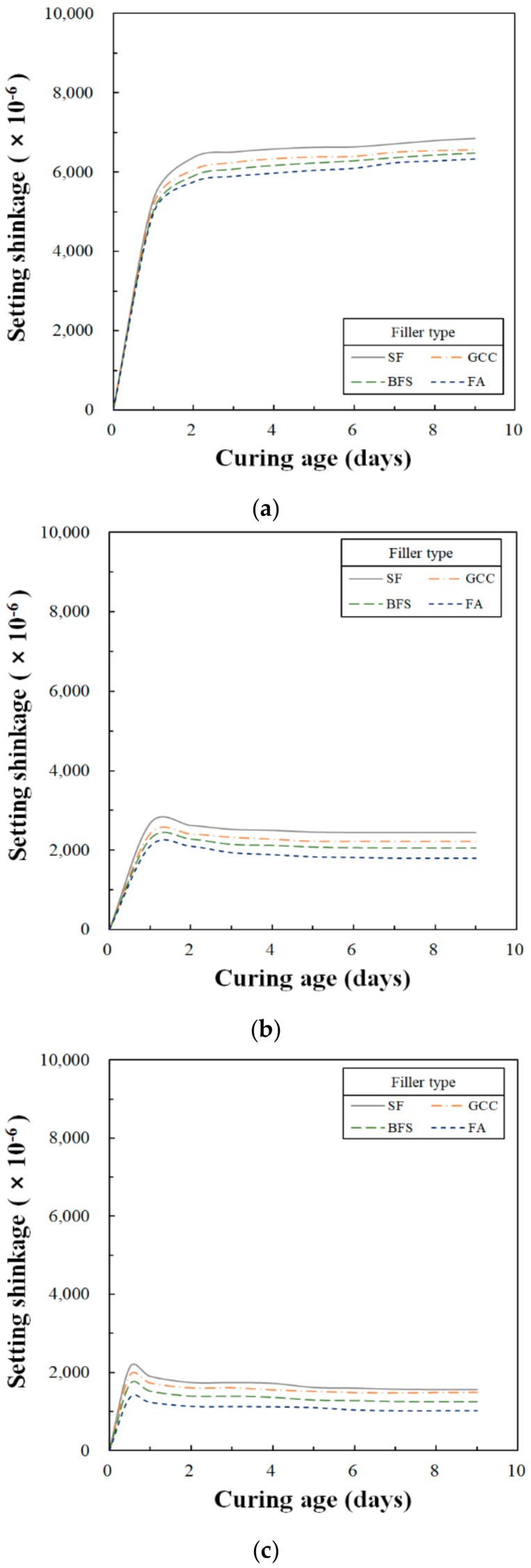
Variation of setting shrinkage strain as a function of curing age for type of filler: (**a**) SRA content is 0%; (**b**) SRA content is 10%; (**c**) SRA content is 15%.

As with the substantial dry shrinkage that is seen in ordinary Portland cement concrete, the setting shrinkage of PC should be considered significant when the material is applied in real structures. Significant setting shrinkage can affect the accuracy of molding or placing, the design of the formwork, and adhesion to the reinforcement or substrate [1]. The setting shrinkage of PC is derived from the volume change resulting from the solidification of liquid polymer resins, regardless of the aggregate properties employed to produce the PC. In other words, it is affected by the type and percentage of polymeric materials. Additionally, a change in dimensional stability can result from crosslinking the polyester resin during the curing process. In contrast, the reduction in the setting shrinkage of PC can be derived from the flow effect produced by using an SRA [27]. The test results of the setting shrinkage of the UP polymer concrete are shown in Figure 4 and Table 8. According to the test result of the setting shrinkage, almost all the setting shrinkage occurred between 12 h and 24 h of the curing age. After that, there was no setting shrinkage.

According to the test results of the setting shrinkage by the SRA contents, the range of the maximum setting shrinkage was from 6333 × 10^−6^ to 6849 × 10^−6^ strain units when the SRA content was 0%, was from 1799 × 10^−6^ to 2445 × 10^−6^ strain units when the SRA content was 10%, and it was from 1020 × 10^−6^ to 1560 × 10^−6^ strain units when the SRA content was 15%. It was identified that the setting shrinkage tended to decrease when the SRA content increased. These test results are similar to what was found by Mani et al. [27], which indicated that the amount of setting shrinkage can be significantly reduced, depending on the composition and quantity of the SRA added to the resin.

According to the test results of the setting shrinkage by filler types, the amount of the setting shrinkage of FA was the smallest, and the amount of the setting shrinkage of SF was the largest. The changes of the setting shrinkage were 516 × 10^−6^ strain units when the SRA content was 0%, was 646 × 10^−6^ strain units when the SRA content was 10%, and finally it was 540 × 10^−6^ strain units when the SRA content was 15%. The differences in setting shrinkage by filler type were not apparent, but a smaller difference in setting shrinkage was identifiable because the particle size mode was smaller.

Conversely, according to the previous study, although the dry shrinkage of ordinary Portland cement mortar and concrete depends upon the aggregate/cement ratio, it ranged from approximately 200 × 10^−6^ to 1200 × 10^−6^ strain units [28]. The setting shrinkage of PC is 5 to 10 times higher than that of ordinary Portland cement concrete, which is 5000 × 10^−6^ to 6000 × 10^−6^ strain units [1]. Comparing these reports with the results of this study shows that the setting shrinkage of PC is substantial, almost the same as the drying shrinkage of ordinary Portland cement concrete when an SRA was added. Therefore, it is necessary to use the appropriate amount of SRA when UP polymer concrete is selected as a repair material and applied to structures of ordinary Portland cement concrete or the manufacture of precast structures.

#### 4.1.2. Thermal Expansion

The coefficient of thermal expansion is defined as the change per unit length when the temperature changes. The thermal shrinkage strain is determined by considering the magnitude of the temperature drop of the concrete and the linear coefficient of thermal expansion at the same time [29]. The coefficient of the thermal expansion of concrete is the sum of the values of the two main constituents’ dissimilar thermal coefficients (i.e., binder and aggregate) [28]. The coefficient of thermal expansion is one of the main parameters of PC that can affect the level of accuracy for dimensional stability [7].

The thermal strain of PC is caused by two parameters: the internal temperature caused by the polymerization reaction of the polymer binder and the external temperature of the atmosphere [30]. If the tensile stress generated by the thermal strain is stronger than the tensile strength of the concrete (i.e., the strength needed to withstand the tensile stress generated by the thermal strain), cracks will occur on the surface of the concrete. A study carried out by O’Connor et al. [15] that compared the maximum thermal and allowable stresses defined in the ACI and AASHTO codes found that the critical tensile stresses defined in ACI and ASSHOTO could be developed in PC overlays located on top of a deck when that deck was fixed by very rigid girders.

**Figure 5:**
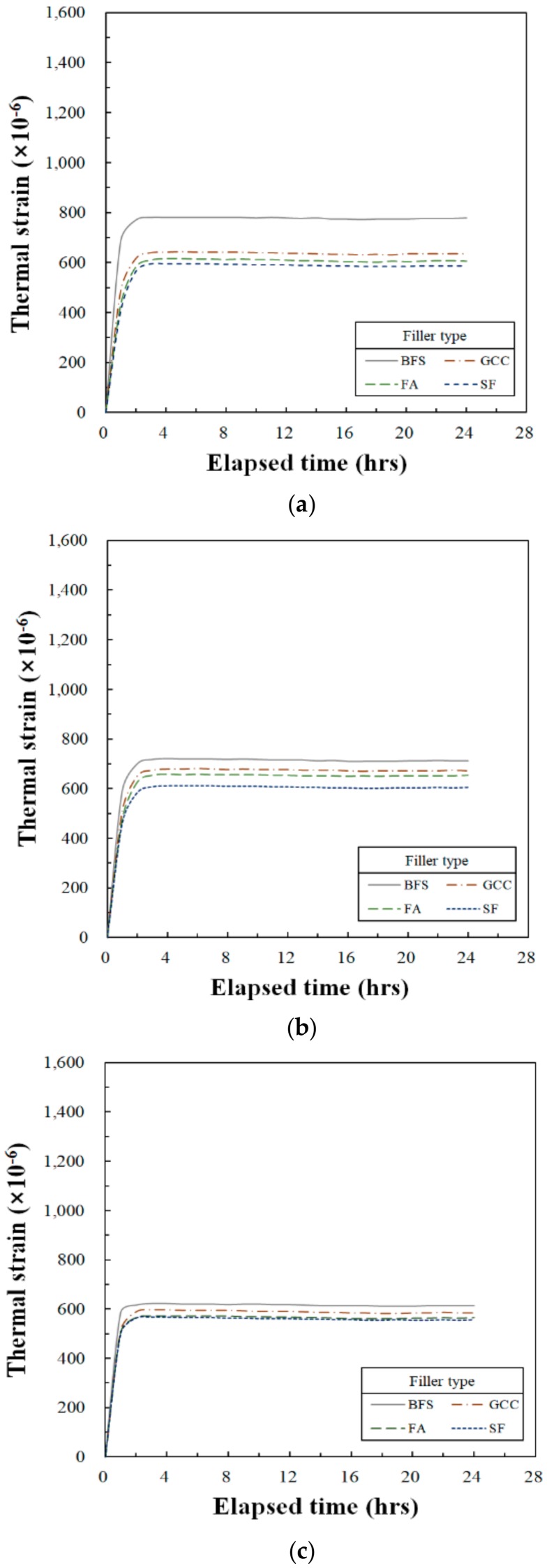
Variation of thermal strain as function of elapsed time for type of filler: (**a**) SRA content is 0%; (**b**) SRA content is 10%; (**c**) SRA content is 15%.

The test results determined in this study regarding the coefficient of the thermal expansion of the UP polymer concrete are shown in Figure 5 and Table 9. This is the result of the thermal strain obtained when the temperature is raised after stabilizing at 25 °C for about 4 h. The changes of the coefficient of thermal expansion by SRA contents are from 11.5 × 10^−6^ to 19.5 × 10^−6^ °C^−1^ at an SRA content of 0%, from 13.1 × 10^−6^ to 16.8 × 10^−6^ °C^−1^ at the SRA content of 10%, and from 14.2 × 10^−6^ to 16.6 × 10^−6^ °C^−1^ when there was an SRA content of 15%. It shows that the coefficient of thermal expansion is decreased when the SRA contents increased. This result shows that the coefficient of thermal expansion also changes depending on the composition and quantity of the SRA added to the resin.

The change in the coefficient of thermal expansion by filler type indicates that the smallest thermal expansion occurred when FA was employed, and the largest when SF was used. The differences of the coefficient of the thermal expansion were 8.0 × 10^−6^ °C^−1^ when the SRA content was 0%, 3.7 × 10^−6^ when the SRA content was 10% and finally 2.4 × 10^−6^ °C^−1^ when the SRA content was 15%. This result shows that the change of the coefficient of the thermal expansion by filler types was decreased when these SRA contents were increased. The cause of these differences by filler type is closely related to the thermal expansion properties of the chemical composition of the filler employed.

Previous studies related to the coefficient of thermal expansion of PC found 23.9 × 10^−6^ °C^−1^ for flexible PC and 13.9 × 10^−6^ °C^−1^ for rigid PC [31]. These values are greater than those of ordinary Portland cement concrete because the coefficient of thermal expansion of the UP resin has a high value of 124 × 10^−6^ °C^−1^ [32].

The stiffness of the polyester–styrene matrix depends upon the chemical composition of the resin. Hence, the more flexible resin with a coefficient of thermal expansion of approximately 23.4 × 10^−6^ °C^−1^ is usually employed for bridge deck overlays. This means that the coefficient of thermal expansion of this PC for the bridge deck overlay was approximately twice as large as that of Portland cement concrete [14,33].

The (approximate) coefficients of linear thermal expansion of cementitious mixtures were: cement paste, 18 × 10^−6^ °C^−1^; mortar, 12 × 10^−6^ °C^−1^; and concrete, ranging from 6 × 10^−6^ to 12 × 10^−6^ °C^−1^ [28]. Also, the majority of the aggregates’ linear thermal expansion coefficients were between approximately 5 × 10^−6^ and 13 × 10^−6^ °C^−1^ [29]. These linear thermal expansion coefficients of concrete and mortar are the result of mixing the cement paste and aggregates.

### 4.2. Stress-Strain Relationship 

**Figure 6:**
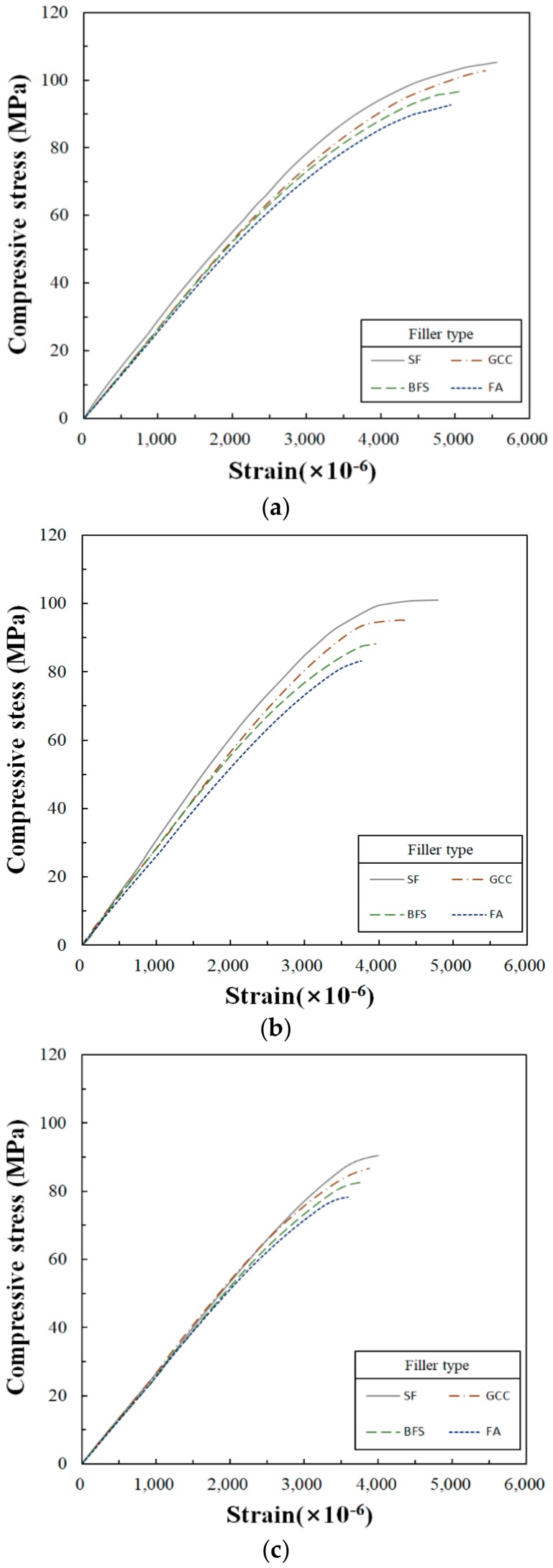
Compressive stress–strain curves by filler types: (**a**) SRA content is 0%; (**b**) SRA content is 10%; (**c**) SRA content is 15%.

Stress–strain curves are the basis for determining the modulus of elasticity. Since concrete is an incomplete elastic material, there are limitations to determining the elastic modulus with this curve. Therefore, the secant modulus of elasticity, also known as the chord modulus, is applied to determine the elastic modulus when the linear elastic section of a material is not clear, such as with concrete [28]. The modulus of elasticity is crucial in analyzing structural behavior. For this study, the modulus of the elasticity was necessary to determine the stress by setting shrinkage and stress by thermal expansion and shrinkage. Inconsistencies in the modulus of elasticity between the material of the existing substrate and repair material are the cause of additional damage when loads are applied parallel to an adhesive layer located between two materials. Since the amount of the deformation of the material with the lower modulus is larger than that of the material with the higher modulus, the loads generated by the deformation are transferred to the higher-modulus materials through the adhesive interface [34]. Thus, the modulus of elasticity is important in maintaining dimensional stability in repair systems. The stress–strain curves of the UP-polymer concrete obtained for this study are shown in Figure 6. These curves are similar to the stress–strain curves of ordinary Portland cement concrete.

#### 4.2.1. Maximum Compressive Strain

The maximum compressive strain is a critical parameter when materials are being selected for a structure’s design. In the present research, the maximum compressive strain was determined based on Table 10. The maximum values determined are shown in Figure 6. First, the effect of an SRA on the change in maximum compressive strain was as follows: strain units ranged from 4940 × 10^−6^ to 5558 × 10^−6^ at 0%, 3765 × 10^−6^ to 4798 × 10^−6^ at 10% and 3595 × 10^−6^ to 4004 × 10^−6^ at 15%. It shows that the compressive strain decreased with increasing SRA content. Here, the compressive strain decreased when the size of the spherical particles employed in the UP polymer concrete was decreased because the small size of the particles contributed to a better packing density and reduced porosity [35].

It was determined that the maximum compressive strain of UP polymer concrete was approximately two to three times higher than that of Portland cement concrete (about 0.002), with the stress at approximately 30 MPa; that of cement paste (about 0.0027) was about 34 MPa [29]. Chandra et al. [1] argued that the substantial strain of PC is influenced by the viscoelastic behavior of the polymer binders.

Also, Table 11 was determined based on Figure 6 when compressive stresses from Figure 6 are regarded as compressive strengths. Compressive strengths developed from 92.7 to 105.2 MPa at 0% SRA, 83.2 to 101.0 MPa at 10% SRA and 78.3 to 90.5 MPa at 15% SRA. It showed that the compressive strength was decreased when the SRA content was increased.

Many studies have investigated the compressive strength of PC. For example, according to the work of Chandra et al. [1], the compressive strength ranged from 80 to 160 MPa. O’Connor et al. [14] reported that the compressive strength developed was 51.7 MPa. Mani et al. [13] indicated that the compressive strength ranged from 41.2 to 68.0 MPa. These compressive strengths were determined using different materials, mix ratios, curing conditions and test methods. The compressive strengths developed in the present study were much higher than those of this earlier body of research.

#### 4.2.2. Modulus of Elasticity

The modulus of elasticity is an important parameter for measuring deformability, not only in PC, but also in other structural materials. The PC employed for overlays of concrete pavement should have a low modulus of elasticity to reduce the shear stresses caused by temperature changes that occur between newly applied overlays and existing concrete substrate [36]. However, construction materials with low modulus values may be disadvantageous when these materials are applied to structures.

The elastic moduli obtained through this study are shown in Table 12, and were determined based on the data found in Figure 6. First, the changes in the moduli of elasticity by SRA contents were from 27.3 × 10^3^ to 30.6 × 10^3^ at 0% of SRA, from 25.8 × 10^3^ to 29.5 × 10^3^ at 10% of SRA and from 22.0 × 10^3^ to 28.9 × 10^3^ at 15% of SRA. It showed that the modulus of elasticity was decreased when the SRA content was increased. This shows that the compressive strengths and the moduli of elasticity were influenced by the applied resin (a binder of UP polymer concrete) and the SRA contents. In terms of the change in the elastic modulus by the filler types, the elastic modulus was the highest when SF was used, and the lowest elastic modulus was when FA was employed. The differences of elastic moduli were 2.5 × 10^3^ at SRA of 0%, 3.7 × 10^3^ at SRA of 10% and 6.9 × 10^3^ MPa at SRA of 15%. As the SRA content increases, the modulus of elasticity tended to increase. In this study, the cause of the difference in elastic modulus by filler type was found to potentially be related to the mode of particle size. However, more research is needed to identify the exact reasons.

There have been many studies on the elastic moduli of polymer and ordinary Portland cement concrete. Among these previous studies, O’Connor [14] found the value to be 17.7 × 10^3^ MPa for polyester–styrene polymer concrete and 25.1 × 10^3^ MPa for ordinary Portland cement concrete. Chandra et al. [1] reported that the modulus of elasticity of polyester polymer concrete ranged from 15 × 10^3^ to 35 × 10^3^ MPa, and the modulus of Portland cement concrete ranged from 20 × 10^3^ to 40 × 10^3^ MPa. Compared to these previous results, it was determined in this research that the elastic modulus of the UP-polymer concrete was similar to the elastic modulus values presented in previous studies. However, the elastic modulus of PC is lower than the elastic modulus of Portland cement concrete, because the elastic modulus of polymer is lower than the elastic modulus of the aggregates. The elastic modulus of PC depends on the elastic modulus of the polymer [37].

**Figure 7:**
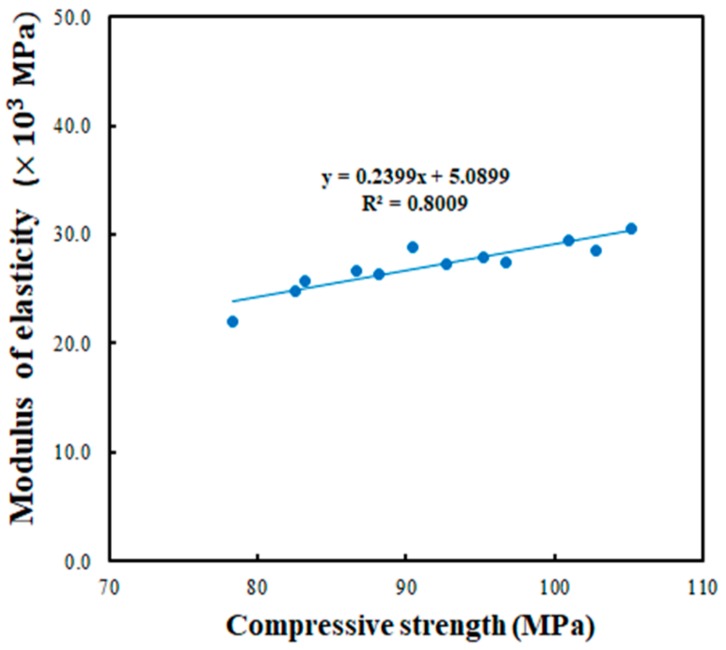
Relation between compressive strength and modulus of elasticity.

The relationship between the compressive strength and modulus of elasticity of all the obtained data regarding UP polymer concrete is shown in Figure 7. From these results, it was determined that the modulus of elasticity increased when the compressive strength was increased. The equation for the compressive strength and modulus of the elasticity is shown below. This equation can be useful to estimate the modulus of elasticity when the compressive strength was determined.

*E_c_* = 0.2399 *f_c_*’+ 5.0899 (1)

Where, *E_c_* is modulus of elasticity of the UP polymer concrete in × 10^3^ MPa and *f_c_*’ is the compressive strength in MPa.

## 5. Conclusions

This study experimentally examined the effects of SRA content and filler type on the deformability of UP polymer concrete in terms of aspects, such as setting shrinkage, thermal expansion, maximum compressive strain, modulus of elasticity, etc.

1) The setting shrinkage was generally stabilized from 12 to 24 h after casting was completed. As the SRA content was increased, the setting shrinkage tended to decrease significantly, and there was no significant difference in setting shrinkage based on filler type.
2) Thermal expansion tended to decrease as the amount of SRA was increased, and the difference in thermal expansion was relatively substantial. Changes in filler produced significant differences depending upon the type used.
3) The maximum compressive strain also tended to decrease as the SRA content was increased, but the difference in compressive strain by the amount of SRA was not significant. There was no significant difference in the compressive strain based on changes in the filler type. As a result, it was determined that the effects of SRA content on setting and thermal shrinkage were not significant.
4) The modulus of elasticity decreased as the SRA content increased. In addition, there were significant differences in the decrease of the moduli of elasticity as the SRA content increased. However, there was no significant difference in the modulus of elasticity by filler types. Also, in this case, the effect of SRA contents on the elastic modulus was greater than the effect of filler types on that.
5) Setting shrinkage was affected by resin, SRA and filler. Additionally, the thermal expansion, maximum compressive strain and modulus of elasticity were affected not only by the resin, SRA, and filler, but also the aggregate. In this study, the effects of SRA content and filler type on setting shrinkage were investigated. As a result, the effect of SRA content on the deformability of UP polymer concrete was found to be more substantial than the effect of filler type.
6) It is desirable to determine the mix proportions, considering both the SRA content and the deformation properties by filler type as determined in this study, in order to secure the proper deformability of UP polymer concrete when it is applied to repair endeavors, structural or nonstructural precast products, etc.
7) The effects of filler type on deformability indicate that: setting shrinkage is influenced by evenly dispersed filler particles; the thermal expansion characteristics of fillers affect thermal expansion; small spherical particles tend to fill porosity, leading to better packing of the aggregate materials and influencing compressive strain; and the modulus of elasticity is influenced by density, which is related to the strength of the filler. Additional research is required to gather greater detail on these topics.

## Figures and Tables

**Table 1:** Properties of the unsaturated polyester (UP) resin.

Specific Gravity (25 °C)	Viscosity (25 °C, mPa·s)	Acid Value	Styrene Content (%)
1.12	300	18.4	40

**Table 2:** Properties of the initiator.

Component	Specific Gravity (25 °C)	Active Oxygen Content (%)	Refractive Index
MEKPO 55% DMP 45%	1.17	9.88	1.4828

**Table 3:** Properties of Styrene-Based shrinkage reduction agent (SRA).

Component	Specific Gravity (25 °C)	Viscosity (25 °C, mPa∙s)	Appearance
Polystyrene 40.6% Styrene monomer 50.4%	1.2	8900	Transparent

**Table 4:** Physical and chemical properties of crystalloid fillers.

Filler	Density (g/cm^3^)	Particle Size (μm)			Chemical Composition (%)					
		Mean	Median	Mode	SiO_2_	Al_2_O_3_	CaO	MgO	Fe_2_O_3_	SO_3_
Ground calcium carbonate (GCC)	2.70	18.18	14.57	28.70	0.62	0.48	53.8	1.21	0.15	0.01
Silica flour (SF)	2.65	31.77	22.74	37.97	98.7	0.52	0.08	0.03	0.18	0.02

**Table 5:** Physical and chemical properties of amorphous fillers.

Filler	Density (g/cm^3^)	Particle Size (μm)			Chemical Composition (%)					
		Mean	Median	Mode	SiO_2_	Al_2_O_3_	CaO	MgO	Fe_2_O_3_	SO_3_
Fly ash (FA)	2.20	22.29	10.75	7.77	49.1	22.7	7.15	1.33	8.31	0.99
Blast furnace slag (BFS)	2.91	13.41	10.10	16.40	29.3	13.3	48.5	2.72	0.53	2.94

**Table 6:** Physical and chemical properties of siliceous aggregate.

Size (mm)	Density (g/cm^3^)	Unit Weight (kg/m^3^)	Fineness Modulus	Water Content (%)	Chemical Composition (%)			
					SiO_2_	Al_2_O_3_	TiO_2_	Fe_2_O_3_
0.2–8.0	2.65	1652	2.81	< 0.1	97.3	1.59	0.02	0.50

**Table 7:** Mix proportion for UP polymer concrete unit: wt.%.

UP resin	SRA	Filler	Aggregate	Initiator (phr)
13.00 (*100*)	0 (*0*)	19	68	(*1*)
11.70 (*90*)	1.30 (*10*)	19	68	(*1*)
11.05 (*85*)	1.95 (*15*)	19	68	(*1*)

Note: UP resin = Unsaturated polyester resin. SRA = Shrinkage-reducing agent; phr = parts per hundred parts of resin.

**Table 8:** Setting shrinkage with SRA contents and filler types.

Filler Types	Setting Shrinkage (×10^−6^)		
	SRA 0%	SRA 10%	SRA 15%
FA	6333	1799	1020
BFS	6480	2056	1248
GCC	6562	2220	1489
SF	6849	2445	1560

**Table 9:** Coefficient of thermal expansion with SRA content and filler type.

Filler Types	Thermal Strain (×10^−6^ °C^−1^)		
	SRA 0%	SRA 10%	SRA 15%
SF	11.5	13.1	14.2
FA	12.3	13.5	14.3
GCC	13.8	14.5	14.5
BFS	19.5	16.8	16.6

**Table 10:** Max. Compressive strains by SRA content and filler types.

Filler Types	Max. Compressive Strain (×10^−6^)		
	SRA 0%	SRA 10%	SRA 15%
FA	4940	3765	3595
BFS	5100	3957	3753
GCC	5403	4400	3880
SF	5558	4798	4004

**Table 11:** Compressive strengths by SRA content and filler types.

Filler Types	Compressive Strength (MPa)		
	SRA 0%	SRA 10%	SRA 15%
FA	92.7	83.2	78.3
BFS	96.7	88.2	82.6
GCC	102.8	95.2	86.7
SF	105.2	101.0	90.5

**Table 12:** Elastic moduli of UP concrete with SRA contents and filler types.

Filler Types	Elastic Modulus (×10^3^ MPa)		
	SRA 0%	SRA 10%	SRA 15%
FA	27.3	25.8	22.0
BFS	27.4	26.4	24.8
GCC	28.5	27.9	26.6
SF	30.6	29.5	28.9
